# Magnetic Attributes of NiFe_2_O_4_ Nanoparticles: Influence of Dysprosium Ions (Dy^3+^) Substitution

**DOI:** 10.3390/nano9060820

**Published:** 2019-05-31

**Authors:** Munirah Abdullah Almessiere, Y. Slimani, H. Güngüneş, S. Ali, A. Manikandan, I. Ercan, A. Baykal, A.V. Trukhanov

**Affiliations:** 1 Department of Biophysics, Institute for Research & Medical Consultations (IRMC), Imam Abdulrahman Bin Faisal University, P.O. Box 1982, Dammam 31441, Saudi Arabia; iercan@iau.edu.sa; 2 Department of Physics, College of Science, Imam Abdulrahman Bin Faisal University, P.O. Box 1982, Dammam 31441, Saudi Arabia; 3 Department of Physics, Hitit University, Çevre Yolu Bulvarı-Çorum 19030, Turkey; gungunes@gmail.com; 4 Mechanical and Energy Engineering Department College of Engineering, Imam Abdulrahman bin Faisal University, P.O. Box 1982, Dammam 31441, Saudi Arabia; sadali@iau.edu.sa; 5 Department of Chemistry, Bharath Institute of Higher Education and Research, Bharath University, Chennai, Tamil Nadu 600073, India; manikandana.che@bharatuniv.ac.in; 6 Department of Nano-Medicine Research, Institute for Research & Medical Consultations (IRMC), Imam Abdulrahman Bin Faisal University, P.O. Box 1982, Dammam 31441, Saudi Arabia; abaykal@iau.edu.sa; 7 Scientific-Practical Materials Research Centre NAS of Belarus, 19 P. Brovki Street, 220072 Minsk, Belarus; truhanov86@mail.ru; 8 Department of Electronic Materials Technology, National University of Science and Technology MISiS, Leninsky Prospekt, 4, Moscow 119049, Russia; 9 Laboratory of Crystal Growth, South Ural State University, Lenin Prospect, 76, Chelyabinsk 454080, Russia

**Keywords:** NiFe_2_O_4_, spinel ferrites, structure, microstructure, magnetization, AC susceptibility

## Abstract

This paper reports the influence of dysprosium ion (Dy^3+^) substitution on the structural and magnetic properties of NiDy*_x_*Fe_2−*x*_O_4_ (0.0 ≤ *x* ≤ 0.1) nanoparticles (NPs) prepared using a hydrothermal method. The structure and morphology of the as-synthesized NPs were characterized via X-ray diffraction (XRD), scanning and transmission electron microscope (SEM, and TEM) analyses. ^57^Fe Mössbauer spectra were recorded to determine the Dy^3+^ content dependent variation in the line width, isomer shift, quadrupole splitting, and hyperfine magnetic fields. Furthermore, the magnetic properties of the prepared NPs were also investigated by zero-field cooled (ZFC) and field cooled (FC) magnetizations and AC susceptibility measurements. The M_ZFC_ (T) results showed a blocking temperature (*T*_B_). Below *T*_B_, the products behave as ferromagnetic (FM) and act superparamagnetic (SPM) above *T*_B_. The M_FC_ (*T*) curves indicated the existence of super-spin glass (SSG) behavior below *T*_s_ (spin-glass freezing temperature). The AC susceptibility measurements confirmed the existence of the two transition temperatures (i.e., *T*_B_ and *T*_s_). Numerous models, e.g., Neel–Arrhenius (N–A), Vogel–Fulcher (V–F), and critical slowing down (CSD), were used to investigate the dynamics of the systems. It was found that the Dy substitution enhanced the magnetic interactions.

## 1. Introduction

Lately, nanosized Ni-spinel ferrites have widely been used in magnetic storage media, magnetic adsorbents, telecommunication, catalysts, microwave absorbers, and computer memories [1,2,3,4,5]. Further, the structural and magnetic characteristics of nanosized Ni-spinel ferrites have been enhanced by substituting varieties of magnetic, nonmagnetic, and rare earths ions [6,7,8]. Such improvement was majorly attributed to the distribution of substitution ions between tetrahedral and octahedral sites of the host and eventual influence on their magnetic moments [8]. Moreover, the use of rare earths as substitution agents in the spinel ferrite was found to cause structural distortion through symmetry preserving the lattice strain and thereby modifying the overall properties of nanosized spinel ferrites [9]. Intense research efforts have been made to enhance the structure, optical, and magnetic properties of rare earth ion substituted nanosized Ni-spinel ferrites [2,3]. Meanwhile, different theories and models have been applied to achieve a better understanding of the unique magnetic properties of these doped nanosized Ni-spinel ferrites [2,3]. Modified magnetic properties of Erbium (Er^3+^) and Samarium (Sm^3+^) substituted nickel ferrites were analyzed by low temperature magnetization, and zero-field cooled and field cooled (ZFC-FC) measurements. Such samples revealed a superparamagnetic (SPM) behavior with very low coercivity (H_c_) and remanence magnetization (M_r_), making them suitable for developing soft magnets [10]. A substitution of Pr, Sm, and La in Ni_0.5_Zn_0.5_Fe_1.95_R_0.05_O_4_ nanoparticles (NPs) was found to modify their magnetic and dielectric properties [11]. Conversely, Cerium (Ce) substituted Ni-Zn at low contents (0.0 ≤ *x* ≤ 0.1) produced high saturation magnetization (M_s_), remanence, and coercivity [12].

So far, studies on Dy^3+^ substituted nanosized Ni spinel ferrites are rare. J. Sahariya et al. [13] reported the temperature dependent spin momentum densities of NiFe_2−*x*_RE*_x_*O_4_ (*x* = 0, 0.05 and RE = Dy, Gd) ferrites measured by using a magnetic Compton spectrometer. The experimental profiles of NiFe_2_O_4_ with doping of Dy and Gd show a similar spin moment to the non-doped sample. The contribution of different constituents in the formation of total spin moment is deduced from the analysis of the Compton line shape. It is seen that Dy^3+^ or Gd^3+^ doping ions at Fe^3+^ sites lead to a redistribution of the spin moment at Fe^3+^ and RE^3+^ sites. The temperature dependent magnetic Compton profiles are decomposed into the constituent profiles of Ni, Fe, Dy/Gd, and diffuse components, arising due to O-2sp states. A decrease in the Fe spin moment from 0.55 ± 0.03 μ_B_/f.u. (in NiFe_2_O_4_) to 0.41(0.50) ± 0.03 μ_B_/f.u. is observed on the partial substitution of Dy(Gd). K. Kamala Bharathi et al. [14] reported a correlation between the microstructure, electrical, and optical properties of Dysprosium-doped nickel-ferrite (NiFe_1.925_Dy_0.075_O_4_) thin films fabricated using sputter-deposition using a stoichiometric bulk target prepared by tsolid-state chemical reaction. Recently, M.A. Almessiere et al. [15] investigated the effects of dysprosium substitution on the structural, microstructural, and magnetic properties of NiFe_2_O_4_ nanoparticles. The formation of the cubic phase of Ni nanosized ferrites was confirmed. The magnetic properties were done by analyzing measurements of the magnetization versus applied field M(H). These measurements were carried out at two different temperatures—room temperature (*T* = 300 K) and low temperature (*T* = 10 K). A noticeable improvement in the differently deduced magnetic parameters, including saturation magnetization (M_s_), remanence (M_r_), and coercivity (H_c_), was observed at both room (*T* = 300 K) and low (*T* = 10 K) temperatures with Dy substitution. The increase in the different magnetic parameters is mainly attributed to the strengthening of A–B exchange interactions owing to the substitution of Fe^3+^ ions with Dy^3^^+^ ions with larger ionic radii, the formation of local strains, and the increase in the magnetic moments (N_B_).

To the best of our knowledge, there is no study reporting the Mossbauer, zero-field cooled (ZFC), and field cooled (FC) magnetizations and AC susceptibility measurements for Dy substituted nanoparticles of Ni spinel ferrites. Accordingly, we examined in the present study the influence of Dy^3+^ doping on the structure, morphology, Mossbauer, ZFC-FC magnetization, and AC susceptibility of NiDy*_x_*Fe_2-*x*_O_4_ (0.0 ≤ *x* ≤ 0.1) nanoparticles. Various products were synthesized using a hydrothermal process.

## 2. Experimental

Various specimens with the chemical formula NiDy*_x_*Fe_2−*x*_O_4_ (0.0 ≤ *x* ≤ 0.1) have been prepared by a hydrothermal process. Analytical grade high purity chemical reagents including Dysprosium III nitrate hydrate (Dy (NO_3_)_3_ H_2_O), Nickel nitrate (Ni(NO_3_)_2_ 6H_2_O), Iron III nitrate (Fe(NO_3_)_3_ 9H_2_O), and Sodium Hydroxide (NaOH) were taken as starting materials. Then, the stoichiometric amount of these compositions was mixed in distilled water and the resulting mixture was left at room temperature. Next, a clear solution was made for pH adjustment using 2M of NaOH solution and placed in a stainless steel-Teflon autoclave to initiate the hydrothermal treatment. Finally, the obtained sample was dried and grinded for further experimental analyses.

A Rigaku Benchtop Miniflex X-ray diffraction (XRD) diffractometer (Tokyo, Japan) with Cu K_α_ radiation at room temperature (RT) over a 2θ range from 20° to 70° was used for the structural analysis. Scanning electron microscopy (SEM, FEI Titan 80–300 kV FEG S/TEM, Hillsboro, OR, USA), along with energy dispersive X-ray spectroscopy (EDX) and transmission electron microscopy (TEM; FEI, Morgagni 268, Prague, Czech Republic), was used for the morphological and composition analyses. AC magnetic susceptibility, zero-field cooled (ZFC), and field cooled (FC) magnetization measurements were performed using a superconducting quantum interference device (PPMS DynaCool, Quantum Design, San Diego, CA, USA). ZFC-FC magnetizations were carried out at a temperature range of 2–400 K under a DC field of 100 Oe. Real and imaginary parts of AC susceptibility measurements were done at temperatures ranging from 350 to 2 K in the presence of an ac applied magnetic field of $H_{\mathrm{ac}}$ = 10 Oe and in different frequencies ranging from 50 to 10,000 Hz. Mössbauer spectra were performed at room temperature using a conventional Mössbauer spectrometer (Fast Com Tec PC-moss II, Oberhaching, Germany) under the constant acceleration mode using ^57^Fe in an Rh matrix with an approximate activity of 10 m Ci. The speed scale and the velocity were calibrated using α-Fe and laser interferometry, respectively. The recorded spectra were analyzed and fit to the inbuilt Win-Normos fitting software (WISSEL company, Duisburg, Germany).

## 3. Results and Discussion

### 3.1. Structure

**Figure 1:**
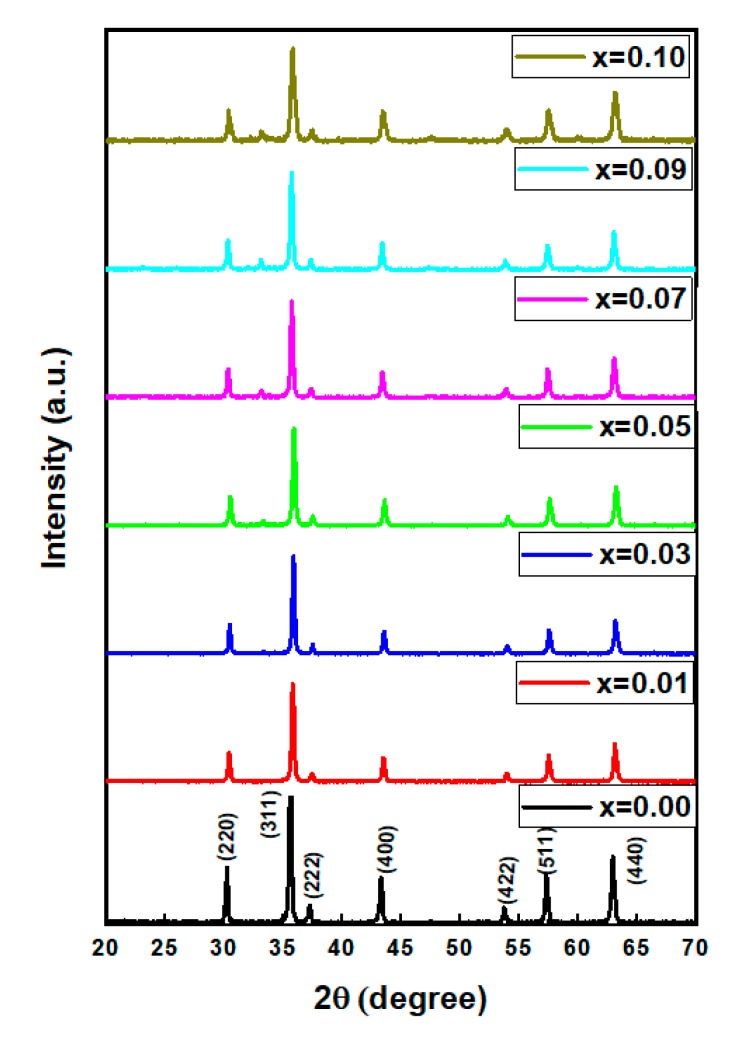
X-ray diffraction (XRD) powder patterns of prepared nanoparticles (NPs).

Figure 1 displays the XRD powder patterns of the studied NiDy*_x_*Fe_2−*x*_O_4_ (0.0 ≤ *x* ≤ 0.1) nanoparticles. Irrespective of Dy^3+^ contents, all the XRD peaks have been verified to the cubic nanosized Ni-spinel ferrites (JCPDS Card Number 54-0964), indicating the lattice site compatibility of the Dy^3+^ ions in the Ni spinel ferrite structure in the absence of any impurity. The calculated structural parameters of the prepared nanosized spinel ferrites were enlists in Table 1. It was realized that lattice parameters elongate due to the increase in the substitution contents and also show ionic radii disparity between Fe^3+^ (0.78 Å) and Dy^3+^ (1.03 Å). The calculated crystallite sizes (D_XRD_) were in the range of 24 to 35 nm [16].

### 3.2. Morphology

**Figure 2:**
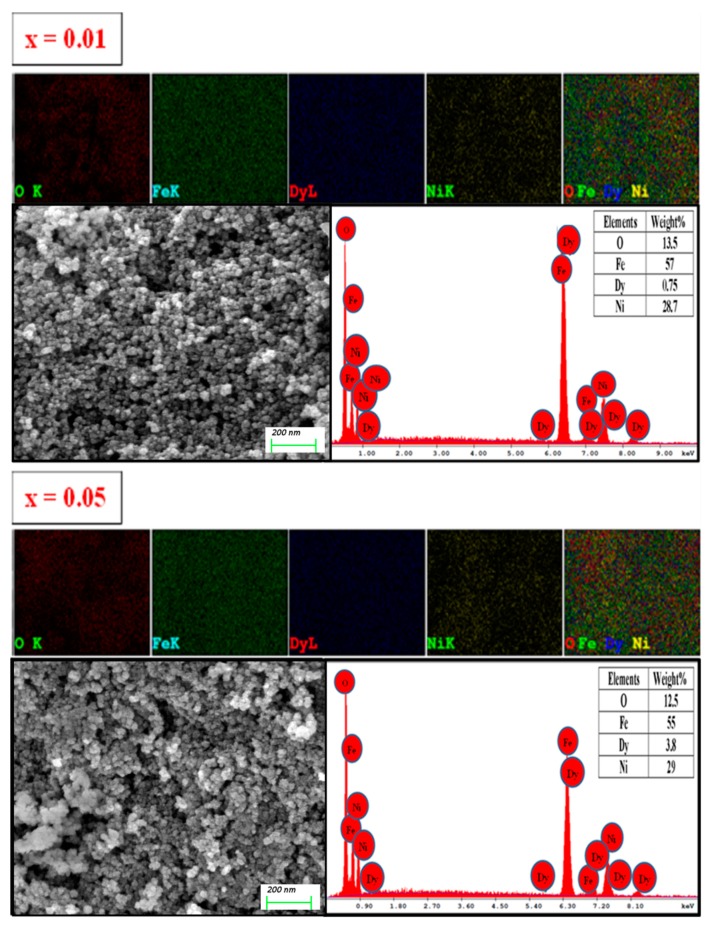
Elemental mapping, Scanning electron microscope (SEM) images and dispersive X-ray spectroscopy (EDX) of two selected *x* = 0.01 and 0.05 NPs.

**Figure 3:**
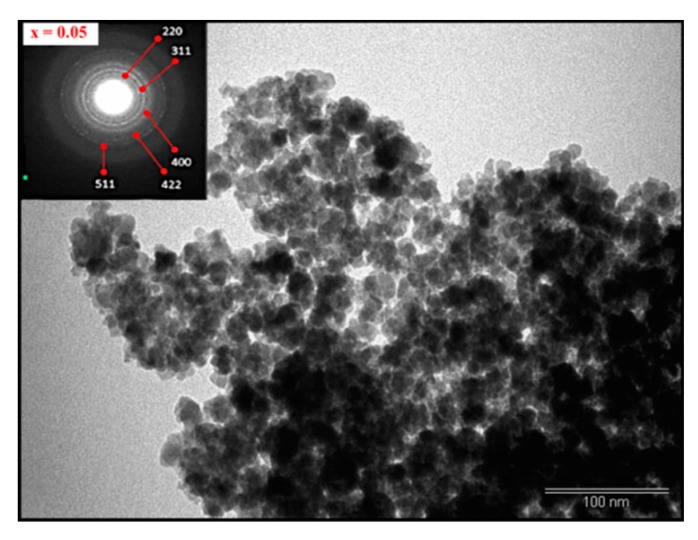
TEM image of selected *x* = 0.05 nanoferrite (Inset: corresponding selected angle electron diffraction (SAED) pattern).

Figure 2 displays the SEM pictures, EDX spectra, and elemental mapping results for *x* = 0.01 and 0.05 samples. The surface morphology of these samples revealed a high degree of nanoparticle agglomeration. EDX analysis proved the existence of appropriate elements (Fe, Ni, Dy, and O) in the samples. Figure 3 illustrates the TEM image of the nanosized spinel ferrites containing Dy^3+^ contents of 0.05. The inset (Figure 3) displays the selected angle electron diffraction (SAED) patterns of the corresponding sample that confirmed the agglomeration of single crystalline Ni spinel ferrite nanoparticles.

### 3.3. Mössbauer Spectra

**Figure 4:**
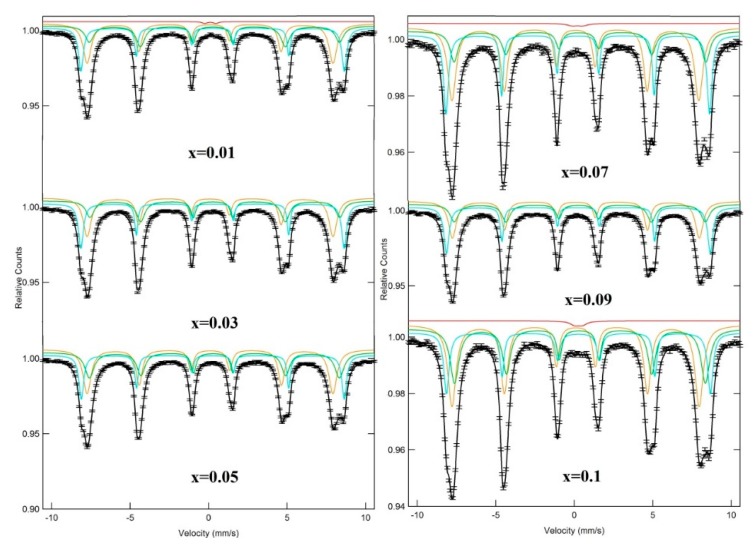
Mössbauer spectra of studied NPs.

Figure 4 depicts the Mössbauer spectra of synthesized NiDy*_x_*Fe_2−*x*_O_4_ (0.0 ≤ *x* ≤ 0.1) NPs at room temperature. Table 2 enlists various Mössbauer parameters calculated by spectral fitting using three sextets (A for the tetrahedral sites and B and B_1_ for the octahedral sites). Fe^3+^ ions in the tetrahedral A site are characterized by a large hyperfine field with an insignificant isomer shift. Conversely, the other two sextets with a comparatively smaller hyperfine field signify the occupation of Fe^3+^ at two dissimilar environments in the B-site [17]. Besides the ferromagnetic sextets, a minute paramagnetic doublet with quadrupole-splitting was evidenced for NiDy_0.01_Fe_1.99_O_4_, NiDy_0.07_Fe_1.93_O_4_, and NiDy_0.1_Fe_1.9_O_4_ NPs. The occurrence of such a paramagnetic doublet was attributed to the fractions of Fe^3+^ with fewer nearest neighbors that possessed magnetically ordered spins. Interestingly, in the spinel ferrite structure, Fe^3+^ did not contribute to the super exchange interaction [18].

The achieved relative area for the A and B sites clearly indicated the occupation of Ni^3+^ in the A and B sites. Kumar et al. acknowledged the preferential occupation of Dy^3+^ in the octahedral B sites of Co-ferrites [19,20]. Thus, the cation distribution in the proposed Dy^3+^ substituted nanoferrites was obtained following the formula unit of (Ni*_y_*Fe_1−*y*_)_A_ (Ni_1−*y*_Dy*_x_*Fe_1+*y*−*x*_)_B_. The distribution of Fe^3+^ over the A and B sites was observed to be relative to the proportional area of A and B in the Mossbauer sub-spectra. Table 2 summarizes the approximate cation distribution obtained from the Mössbauer spectra. The results in Table 2 revealed that Fe^3+^ cations emigrated from the B site to A site due to Dy^3+^ substitution. The line width of the A site was randomly altered, whereas for B and B_1_ sites, it was enhanced with the substitution of Fe^3+^ (0.64 Å) in the B sites by Dy^3+^ (0.91Å) having larger ionic radii than the former one. This observation authenticated the increase in the degree of disorder due to the substitution on B sites. 

The values of the hyperfine magnetic fields for the A and B sites (Table 2) in the studied nanosized spinel ferrites were first reduced with an increase in Dy^3+^ contents up to 0.05 and then enhanced at 0.07. Eventually, the hyperfine field for A site was continuously enhanced, but for the B site it was diminished. This alteration in the hyperfine field for the A and B sites was attributed to the addition of the diamagnetic Dy^3+^ that replaced the ferromagnetic Fe^3+^ with a higher magnetic moment (5 µ_B_) and lowered the average number of magnetic linkages (${\mathrm{Fe}}_{A}^{3+}-O-{\mathrm{Fe}}_{B}^{3+}$). Thus, Fe^3+^ nuclei experienced a reduction in the magnetic field at both the sublattices up to the Dy3+ content of 0.05. Beyond 0.05, the number of Fe^3+^ at the A site was augmented, thereby increasing the hyperfine magnetic field and magnetic moment of Fe^3+^ at the A site.

### 3.4. ZFC-FC Magnetizations

**Figure 5:**
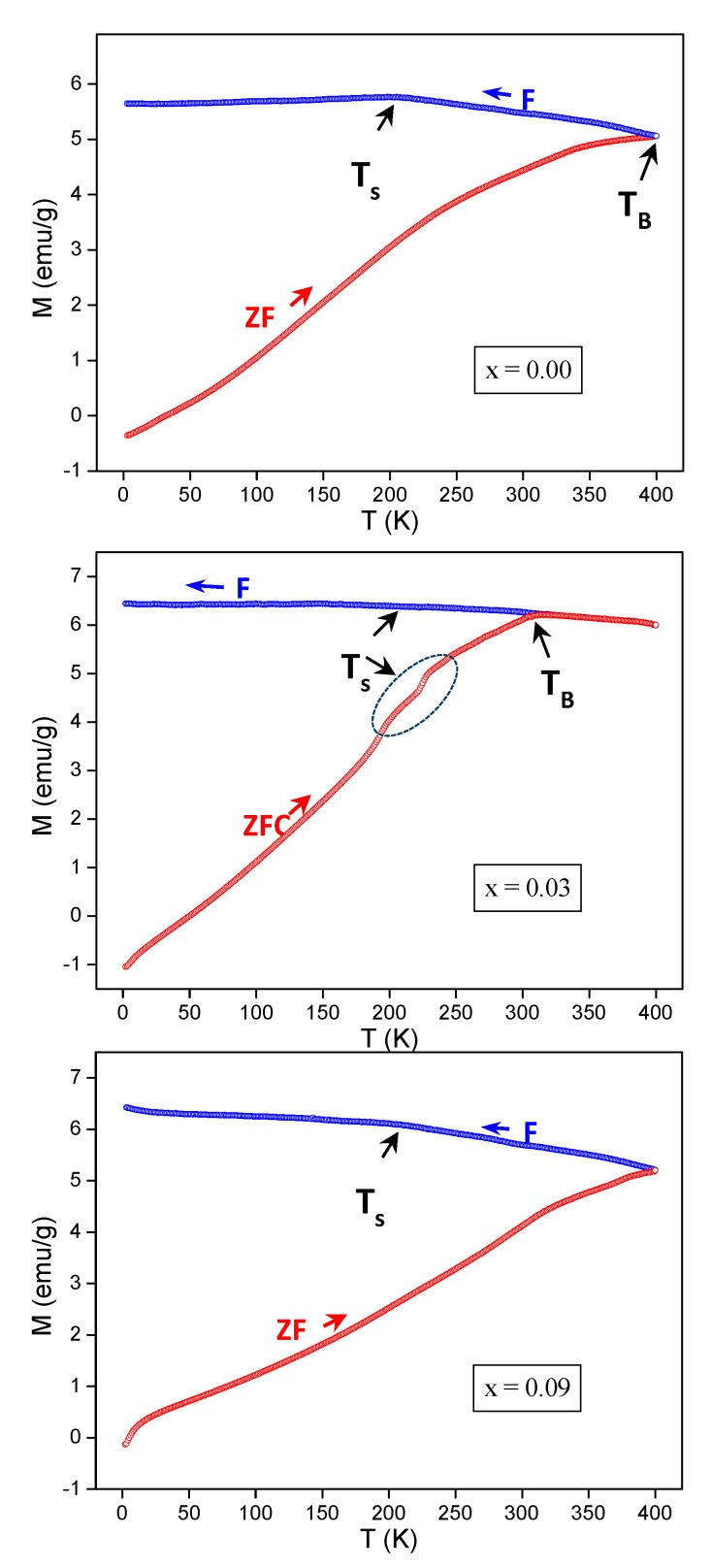
M_ZFC_ (T) and M_FC_ (T) curves of the three selected NPs.

Figure 5 shows the curves of zero-field-cooled (ZFC) and field-cooled (FC) temperature dependencies of the magnetization, M_ZFC_ (*T*) and M_FC_ (*T*), of NiFe_2−*x*_Dy*_x_*O_4_ (where *x* = 0.00, 0.03 and 0.09) NPs. These measurements were performed in a temperature interval ranging between 2 and 400 K under a DC field of 100 Oe. For M_ZFC_ (*T*) measurements, the sample was cooled, first of all, from room temperature (RT) to a very low temperature in the absence of an applied field and subsequently the magnetization was recorded by increasing the temperature in the presence of the field. However, in the M_FC_ (*T*) measurements, the magnetization was recorded by cooling the product in the presence of applied field. A splitting and a large irreversibility between M_ZF_ (*T*) and M_FC_ (*T*) curves for different synthesized products can be clearly seen in Figure 5. The M_FC_ (*T*) increased gradually and remained constant below temperature $T_{s}$, while the M_ZFC_ (*T*) decreased with a lowering of the temperature down to about 4 K. The dispersion of M_ZFC_-M_FC_ versus *T* curves is congruent with the poly-disperse character of magnetic NPs, with a correlated distribution in particle size and individual anisotropy axes [21]. The enlargement could also owe to dipolar interactions among particles [21].

It is reported in the literature that the manifestation of a peak in the M_ZFC_ (T) plots is associated to the blocking temperature (*T*_B_) [22]. The curves of M_ZFC_ (T) of the prepared products showed an incomplete maximum or broad maximum at around the temperature noted *T*_B_. This is typical for superparamagnet (SPM) materials, which show the properties of classical paramagnet materials (PM) above *T*_B_, where the total spin is equal to the spin of a whole NPs but behave as ferromagnetic (FM) materials below their blocking temperature (*T*_B_). Below *T*_B_, the M_FC_ (*T*) and M_ZFC_ (*T*) curves considerably diverge, and the various ferrite NPs are in the FM state (blocked state). Above *T*_B_, the M_FC_ (*T*) and M_ZFC_ (*T*) curves coincide, which is because all NPs are in the same SPM state. At *T* = *T*_B_, the thermal activation overcomes the magnetic anisotropy barrier, which leads to fluctuations in magnetization [23]. Therefore, the wide peak at *T*_B_ in the M_ZFC_ (*T*) curves is an indication of a broadened energy barrier distribution. Further, it can be seen that the blocking temperature varies by increasing Dy substitution content. The non-substituted product NiFe_2_O_4_ shows a blocking temperature around *T*_B_ ≈ 390 K. The *x* = 0.03 product exhibits a well-defined *T*_B_ at around 300 K. By further increasing the amount of Dy, the product synthesized with *x* = 0.09 was not able to reach the *T*_B_ within 400 K, so the *T*_B_ value is superior than 400 K for *x* = 0.09. It can be seen clearly that the blocking temperature decreases for a lower Dy content (*x* = 0.03) and then increases for higher content (*x* = 0.09). The dependence of the *T*_B_ on particles size has been reported in previous studies [24]. The lower *T*_B_ is attributed to smaller particle size or narrow size distribution. However, the higher *T*_B_ represents a larger particle size. Nevertheless, the different *x* = 0.00, 0.03 and 0.09 products show approximately same particles size. Therefore, the variations in blocking temperature with substitution effects are not predominantly influenced by the grain size. Thus, in addition to the particle size effect, the *T*_B_ could also be affected by numerous other extrinsic factors, mostly related to interactions among particles and intrinsic factors that principally include a magneto–crystalline, surface and shape anisotropy [22,25]. We noticed in the synthesized product with *x* = 0.03 that the M_ZFC_ (*T*) exhibits a breaking at temperatures indicated by the dashed circle in Figure 5.

On the other hand, the M_FC_ (*T*) curves increase smoothly for different samples with a decrease of temperature, while a kind of saturation in the magnetization is noticed below the temperature noted by $T_{s}$ for all samples. It is reported in the literature that for SPM nanoparticles, the curve of M_FC_ (*T*) increases continuously [26,27]. Nevertheless, in the case of super-spin glass (SSG) systems in which the interactions among particles are strong, a flat type or a slow increase is observed [26,27]. Therefore, the detected flat nature below $T_{s}$ in the M_FC_ (*T*) curves establishes the occurrence of an SSG-like state. The origin of the observed magnetic features in ZFC-FC magnetization part will be discussed in detail in the analyses of the AC susceptibility measurements. The latter are a useful way to identify the freezing dynamics of the spin-glass (SG) materials.

### 3.5. AC Susceptibility 

For measurements of AC susceptibility ($\chi_{\mathrm{ac}}$), an AC magnetic field ($H_{\mathrm{ac}}$) is applied to the sample and, as a consequence, a resultant magnetic moment is measured. The $\chi_{\mathrm{ac}}$ is represented as follows:

$$\chi_{\mathrm{ac}}=\chi^{\prime }+i\chi^{\prime \prime }$$

where *χ*’ real and $\chi^{\prime \prime }$ imaginary parts are, respectively, the in-phase and out-phase components of $\chi_{\mathrm{ac}}$. It should be noted that the relaxation time (τ) of the AC susceptibility measurement is not based upon the energy barrier ($E_{a}=K_{\mathrm{eff}}V$ where $K_{\mathrm{eff}}$ is the effective anisotropy constant and V is the volume of particles). However, it is influenced by the external excitation frequency. The AC susceptibility measurements give important details about the dynamics of the systems and the strength of exchange interactions between the magnetic nano-particles (MNPs) and between the different cations. 

**Figure 6:**
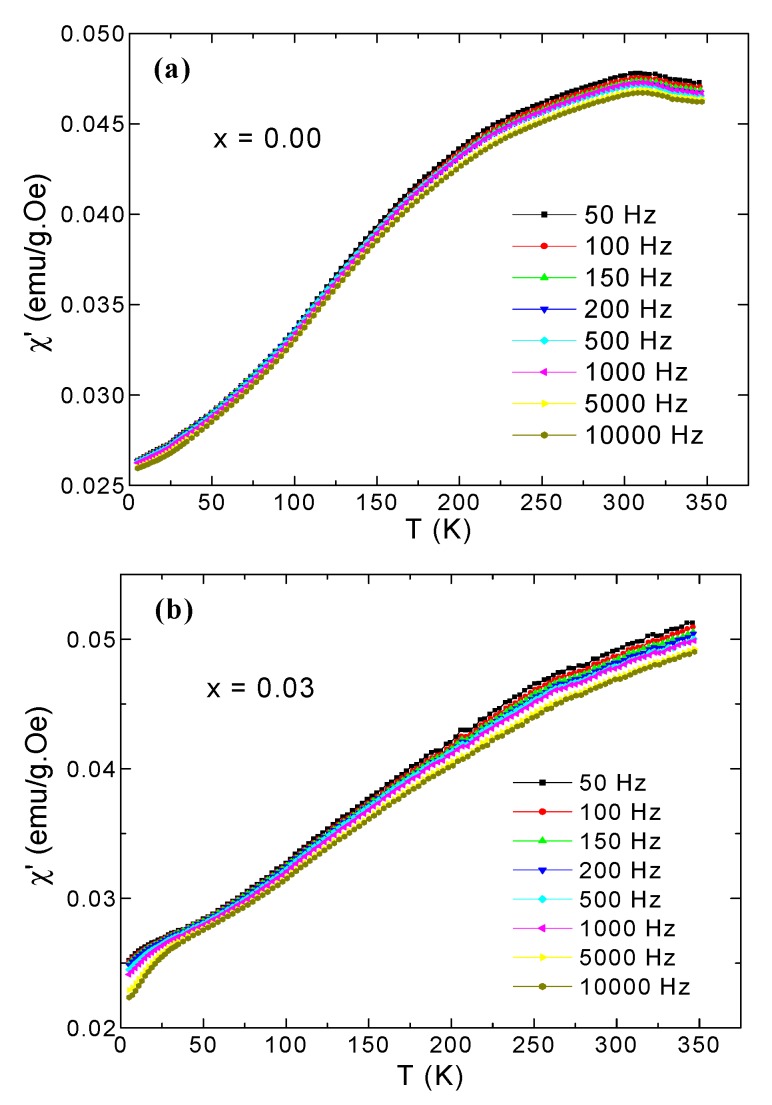
*χ*’(*T*) curves of the prepared (**a**) NiFe_2_O_4_ and (**b**) NiFe_1.97_Dy_0.03_O_4_ NPs.

Firstly, we will discuss the *χ*’ real part measurements of the two NPs samples with *x* = 0.00 and 0.03. Figure 6 presents the curves of *χ*’versus *T*, ranging from 350 to 2 K, for *x* = 0.00 and 0.03 products, performed in the presence of an $H_{\mathrm{ac}}$ = 10 Oe and in a frequency range of 50–10^4^ Hz. The magnitude of *χ*’ for *x* = 0.03 increased slightly compared to the non-substituted product (*x* = 0.00), which is in accordance with M_ZFC_ (T) and M_FC_ (T) measurements. The in-phase AC susceptibility data of the different samples showed dispersion and a decrease in magnitude while increasing the applied frequency from 50 Hz to 10 kHz. The *χ*’(*T*) curve of the non-substituted NiFe_2_O_4_ NPs exhibited a peak at around 300 K. However, the *x* = 0.03 product did not show any peak up to 350 K.

**Figure 7:**
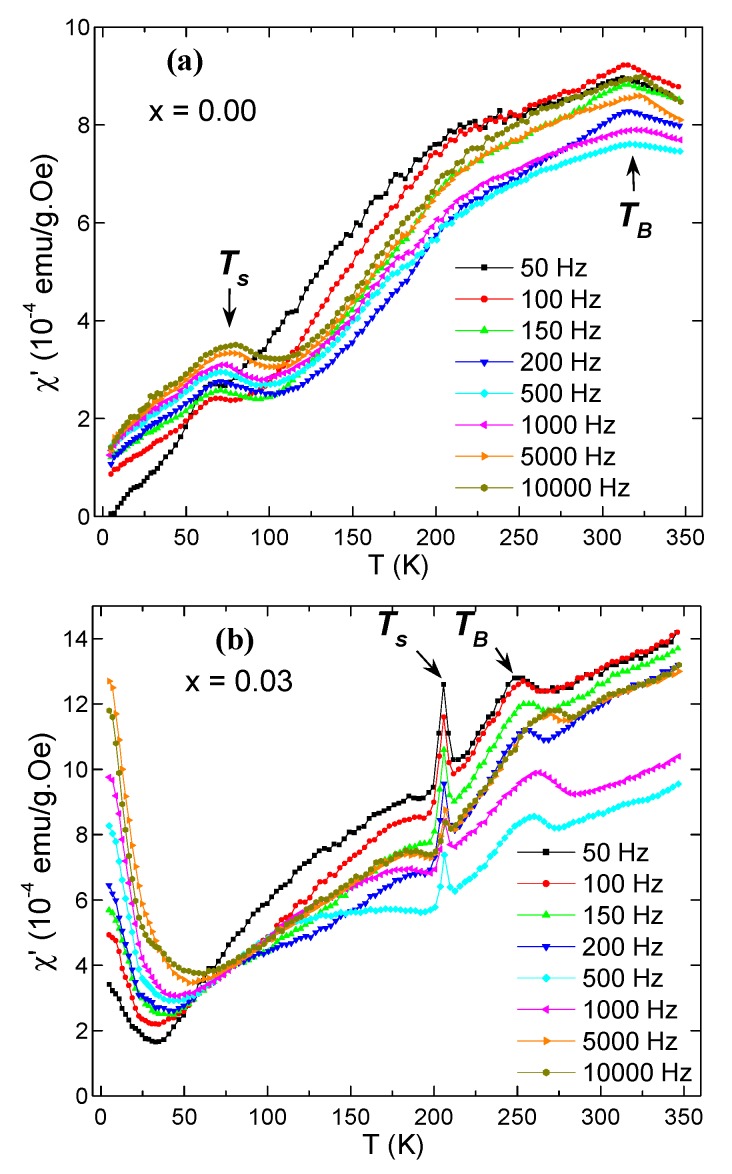
χ″(T) curves for (**a**) NiFe_2_O_4_ and (**b**) NiFe_1.97_Dy_0.03_O_4_ NPs.

Figure 7 shows the $\chi^{\prime \prime }\left(T\right)$ curves for NiFe_2-*x*_Dy*_x_*O_4_ (where *x* = 0.00 and 0.03) performed in a $H_{\mathrm{ac}}$ = 10 Oe and in the frequency range of 50–10^4^ Hz. It can be seen that both samples display two peaks—the first at the higher temperature indicated by *T*_B_ in the figure, which is associated with magnetic blocking of huge core spins, and the second indicated by $T_{s}$, which can be associated with the spin-glass freezing on the surface of a single NP [28]. The *χ*’(*T*) curves do not offer any information about these two peaks, hence from now on we will focus only on analyses of $\chi^{\prime \prime }\left(T\right)$ curves.

At the same applied frequency, the *T*_B_ and $T_{s}$ shifted to lower temperatures with Dy substitution compared to the non-substituted one. This is consistent with the M_ZFC_ analyses. Both the blocking temperature *T*_B_ and spin-glass freezing temperature $T_{s}$ are affected by frequency. Both show a shift to higher temperatures upon increasing the value of the applied frequency (f). Similar behavior is observed in the spin-frustrated system of CoFe_2_O_4_ NPs dispersed in an SiO_2_ matrix [28]. The shifting with *f* is helpful for evaluating dynamic magnetic behaviors, deducing the anisotropic energy, the magnetic anisotropy, and the interaction strength between MNPs.

**Figure 8:**
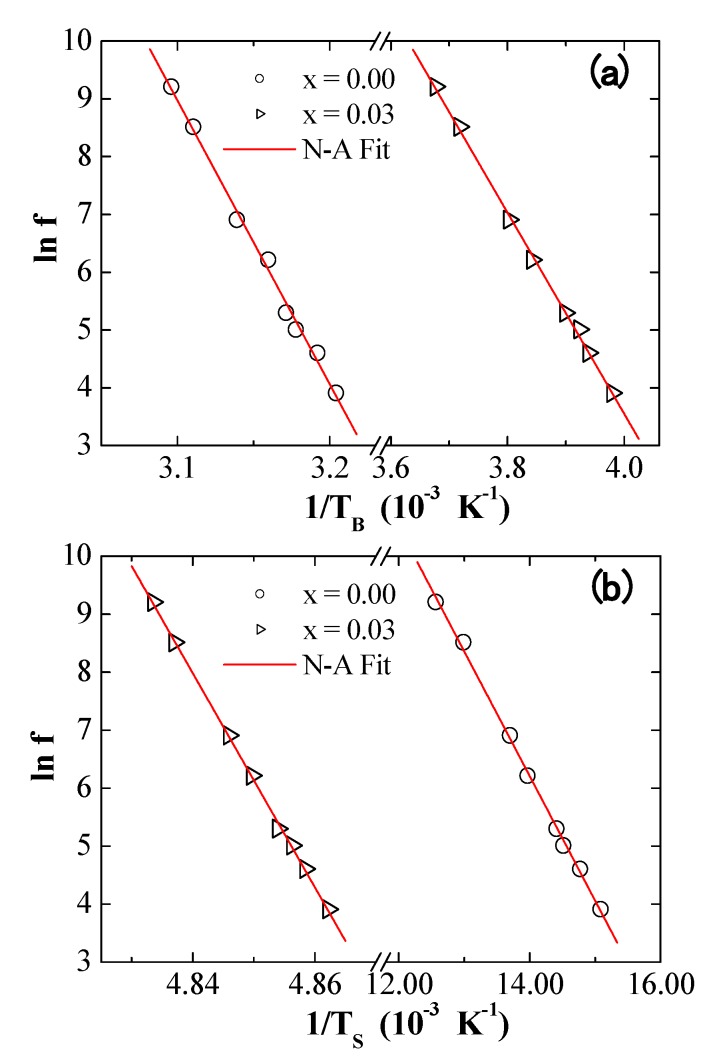
Plots of (**a**) ln(f) versus $1/T_{B}$ and (**b**) ln(f) versus $1/T_{s}$ for two selected NiFe_2−*x*_Dy*_x_*O_4_ NPs where *x* = 0.00 and 0.03 fit to the Neel–Arrhenius (N–A law) (solid lines).

Various physical laws can be used to investigate the f-dependence shift of *T*_B_ and $T_{s}$ temperatures. The Neel–Arrhenius (N–A) law was first tested to fit the experimental data (Figure 8). This theory is valid for thermal excitations of non-interacting single-barrier NPs and is expressed as follows [29,30]:

$$\tau =\tau_{o}\;exp\left(E_{a}/k_{B}T\right)$$

where τ=1/f is the measured time, $\tau_{0}$ is the jump attempt time (in the range of 10^−9^–10^−^^13^ s), $k_{B}$ is the Boltzmann constant, and $E_{a}=K_{\mathrm{eff}}V$ is the activation energy barrier. The estimated values of $\tau_{0}=1/f_{0}$, $E_{a}/k_{B}$ and $K_{\mathrm{eff}}$ for different samples are given in Table 3. The best N–A fit offers very unreasonable values for $\tau_{0}$ and $E_{a}/k_{B}$. This indicates that the synthesized products do not obey the thermally activated N–A law and, as a consequence, they are non-interacting.

The Vogel–Fulcher (V–F) law is a useful model for investigating the interactions between NPs. This law uses an additional parameter, $T_{0}$, that represents the strength of inter-particle interactions. Based on this model, the relaxation is described as follows [29,30]:

$$\tau =\tau_{o}\;\exp\left[E_{a}/k_{B}(T-T_{0}\right)].$$

**Figure 9:**
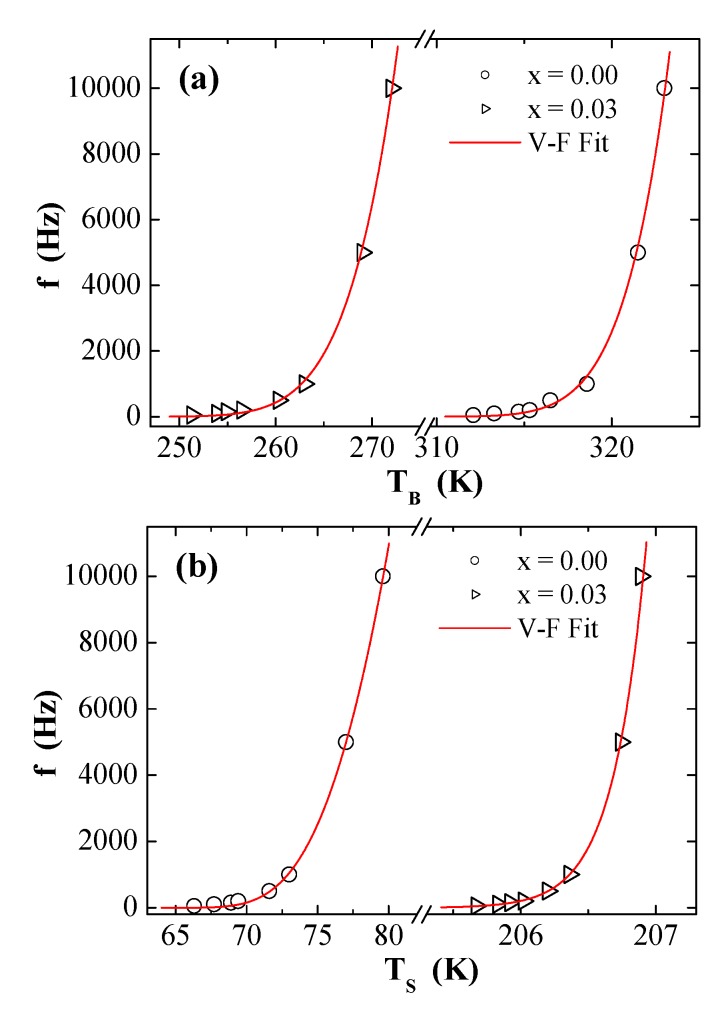
Plots of (**a**) *f* versus $T_{B}$ and (**b**) *f* versus $T_{s}$ for NiFe_2−*x*_Dy*_x_*O_4_ (*x* = 0.00 and 0.03) NPs (solid lines present the Vogel–Fulcher (V–F) fit).

The fitting data using the V–F law of the plots of f vs. *T*_B_ and f vs. $T_{s}$ for the prepared products are illustrated in Figure 9a,b, respectively. The different estimated parameters are summarized in Table 3. The analysis of f-dependent *T*_B_ now gives reasonable $\tau_{0}$ and $E_{a}/k_{B}$ values. Obviously, the *T*_0_ values are not negligible compared to *T*_B_. The occurrence of *T*_0_ confirms the presence of moderate inter-particle interactions between the NPs [28,29,30]. It is found, moreover, that $\tau_{0}$ increased more for the *x* = 0.03 product than for the x = 0.00 one. The increase in the $\tau_{0}$ for *x* = 0.03 product suggests the strengthening of interactions between NPs [29,30]. Compared to the *x* = 0.00 product, the values of $E_{a}/k_{B}$ and $K_{\mathrm{eff}}$ improved with Dy substitution for *x* = 0.03. This improvement iresulted from the strengthening of magnetic interactions among different NPs and the increase of magnetic anisotropy sources [29,30].

**Figure 10:**
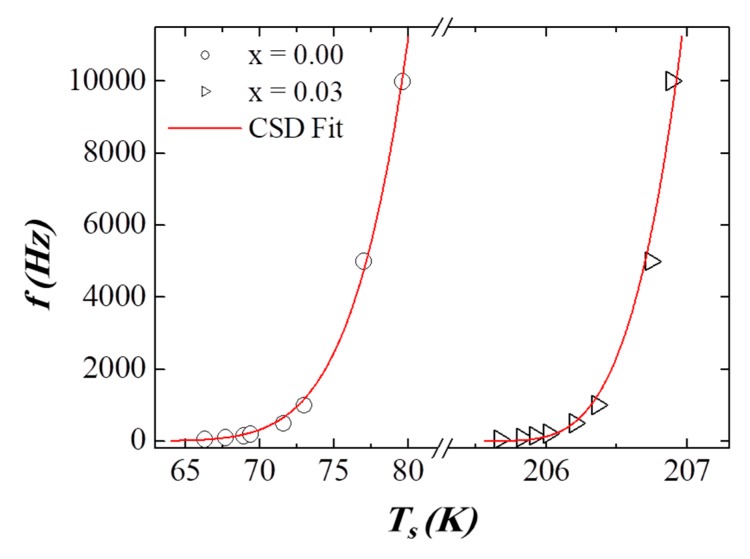
Plots of *f* versus $T_{s}$ for the two selected *x* = 0.00 and 0.03 NPs. The solid lines represent the critical slowing down (CSD) fit.

In other hand, the investigation of *f* vs. $T_{s}$ provides unphysical values for $\tau_{0}$. Therefore, the critical slowing down (CSD) law is used to study the presence of SG behavior in the synthesized NPs. Based on this model, the relaxation is expressed as [30]:

$$\tau =\tau_{0}^{*}{\left[\frac{T_{s}}{T_{g}}-1\right]}^{-\left(z\upsilon \right)}$$

where $\tau_{0}^{*}$ is associated to the coherence time of coupled individual “atomic” spins in the NP (in the range 10^−6^–10^−13^ s) [31], $T_{g}$ is the SG freezing temperature, and $T_{s}$ is the *f*-dependent freezing temperature. The “zυ” is the critical exponent that offers information about the SG, and it varies from 4 to 12 for various SG systems [28]. We fit the same *f* vs. $T_{s}$ data using the CSD law, in order to examine the possibility of the SG nature (Figure 10). The various deduced parameters are listed in Table 3. The obtained reasonable values of $\tau_{0}$, $T_{g}$ and “zυ” proved the existence of SG behavior in the prepared samples. Similar comportment has been reported in numerous products, such as CoFe_2_O_4_/(SiO_2_)*_x_* systems [28], Fe_3_O_4_ MNPs (zυ=8.2 and $\tau \;\sim \;10^{-9}\;s$) [32,33], soft ferrite Ni_0.3_Zn_0.7_Fe_2_O_4_ NPs (zυ=8.01 and $\tau \;\sim \;10^{-12}\;s$) [34], and La_0.9_Sr_0.1_MnO_3_ NPs [35]. It is reported that the strength of magnetic interactions increases based on the decreasing “zυ” exponent. The non-substituted NiFe_2_O_4_ product exhibits a “zυ” value equal to 5.11, and it decreases to 3.95 with Dy substitution for *x* = 0.03. This result indicates the improvement of the magnetic interactions among NPs for the *x* = 0.03 product.

## 4. Conclusions

Series of Dy^3+^ doped ferrite nanoparticles of NiFe_2-*x*_Dy*_x_*O_4_ (0.0 ≤ *x* ≤ 0.1) NPs were prepared using a hydrothermal method. The as-prepared specimens were thoroughly characterized using various analytical measurement techniques to determine their structure, morphology, and distinct magnetic traits. An XRD pattern of nanoferrites confirmed the formation of a spinel cubic structure. The Mössbauer spectra displayed the cation distribution, verifying the occupation of Dy^3+^ ions at octahedral B sites. The measurements of ZFC-FC magnetization and AC susceptibility were examined. The magnetic data showed the existence of two critical temperatures. The first one is the blocking temperature $T_{B}$, which corresponds to the magnetic blocking of huge core spins, and the second is $T_{s}$, which can be associated to spin-glass freezing arising on the surface of a single NP. The $T_{B}$ and $T_{s}$ temperatures vary with the Dy substitution. This is due to numerous intrinsic and extrinsic factors that principally include magneto-crystalline factors, interactions among particles, surface, and shape anisotropy. Furthermore, both the $T_{B}$ and $T_{s}$ temperatures are affected by applied frequency. Various physical laws, such as Neel-Arrhenius, Vogel-Fulcher, and critical slowing down, are used to investigate the f-dependence shift of the $T_{B}$ and $T_{s}$ temperatures. It was found that the Dy substitution enhances the magnetic interactions. Compared to the *x* = 0.00 product, the $E_{a}/k_{B}$ and $K_{\mathrm{eff}}$ values improved for *x* = 0.03. This proved that the magnetic interactions are enhanced due to the Dy substitution.

## Figures and Tables

**Table 1:** Structural parameters of studied NiDy*_x_*Fe_2−*x*_O_4_ NPs.

*x*	a (Å)	V (Å^3^)	D_XRD_ (nm)
0.00	8.3378	579.6451	34.7
0.01	8.3397	580.0333	33.1
0.03	8.3413	580.3630	34.7
0.05	8.3424	580.3463	30.8
0.07	8.3473	579.3824	32.3
0.09	8.3470	579.9142	33.1
0.10	8.3473	580.9998	24.3

**Table 2:** Evaluated Mössbauer parameters of studied ferrite NPs (B_hf_: hyperfine magnetic field, I.S.: isomer shift, Q.S.: quadrupole splitting, W: line width, and R_A_: Relative area).

*x*	Assignment of Sites	I.S. (±0.002) (mm/s)	Q.S. (±0.004) (mm/s)	B_hf_ (±0.02) (T)	W (±0.01) (mm/s)	R_A_ (%)	Cation Distribution
0.01	Sx-A: Fe^3+^	0.368	0.005	52.045	0.218	17.298	(Ni_0.65_Fe_0.35_)_A_ [Ni_0.35_ Dy_0.01_Fe_1.64_]_B_O_4_
	Sx-B: Fe^3+^	0.438	0.097	49.407	0.401	35.575	
	Sx-B_1_: Fe^3+^	0.202	0.011	48.566	0.37	46.375	
	Db: Fe^+3+^	0.226	0.717	−	0.399	0.752	
0.03	Sx-A: Fe^3+^	0.364	0	52.009	0.205	17.358	(Ni_0.66_Fe_0.34_)_A_ [Ni_0.34_Dy_0.03_Fe_1.63_]_B_O_4_
	Sx-B: Fe^3+^	0.455	0.119	49.337	0.383	34.389	
	Sx-B_1_: Fe^3+^	0.204	0.09	48.55	0.38	48.253	
0.05	Sx-A: Fe^3+^	0.365	0.008	51.941	0.257	16.783	(Ni_0.67_Fe_0.33_)_A_ [Ni_0.33_Dy_0.05_Fe_1.62_]_B_O_4_
	Sx-B: Fe^3+^	0.438	0.071	49.379	0.395	35.528	
	Sx-B_1_: Fe^3+^	0.231	−0.018	48.559	0.402	47.689	
0.07	Sx-A: Fe^3+^	0.366	−0.008	52.057	0.242	17.866	(Ni_0.65_Fe_0.35_)_A_ [Ni_0.35_Dy_0.07_Fe_1.58_]_B_O_4_
	Sx-B: Fe^3+^	0.464	0.072	49.737	0.466	25.675	
	Sx-B_1_: Fe^3+^	0.218	−0.023	48.76	0.422	54.952	
	Db: Fe^3+^	0.345	0.625	−	0.725	1.5067	
0.09	Sx-A: Fe^3+^	0.365	0.004	52.119	0.219	19.981	(Ni_0.62_Fe_0.38_)_A_ [Ni_0.38_Dy_0.09_Fe_1.53_]_B_O_4_
	Sx-B: Fe^3+^	0.424	0.066	49.614	0.445	35.589	
	Sx-B_1_: Fe^3+^	0.209	−0.005	48.766	0.394	45.43	
0.10	Sx-A: Fe^3+^	0.37	−0.004	52.123	0.239	20.331	(Ni_0.6_Fe_0.4_)_A_ [Ni_0.4_Dy_0.1_Fe_1.5_]_B_O_4_
	Sx-B: Fe^3+^	0.441	0.053	49.544	0.397	31.53	
	Sx-B_1_: Fe^3+^	0.198	−0.02	48.825	0.413	46.22	
	Db: Fe^3+^	0.379	0.481	−	0.696	1.9186	

**Table 3:** Fitting parameters ($\tau_{o}$, $E_{0}/k_{B}$ and ) of prepared NiFe_2−*x*_Dy*_x_*O_4_ (*x* = 0.00 and 0.03) NPs estimated using different laws.

Models	Parameters	Values			
		Peak (T_B_)		Peak (T_S_)	
		*x* = 0.00	*x* = 0.03	*x* = 0.00	*x* = 0.03
**Neel–Arrhenius**	$\tau_{o}$ (s)	1.05 × 10^−70^	1.68 × 10^−32^	1.69 × 10^−16^	1.26 × 10^−392^
	$E_{0}/k_{B}$ (K)	49,083	17,403	2150	184,793
	$K_{\mathrm{eff}}$ (erg/cm^3^)	3.09 × 10^5^	1.09 × 10^5^	1.35 × 10^4^	1.16 × 10^6^
**Vogel–Fulcher**	$\tau_{o}$ (s)	5.45 × 10^−10^	1.32 × 10^−9^	8.00 × 10^−7^	6.06 × 10^−46^
	$E_{0}/k_{B}$ (K)	367.57	621.53	100.38	2188.95
	$T_{o}$ (K)	292.71	216.76	58.82	183.84
	$K_{\mathrm{eff}}$ (erg/cm^3^)	2.32 × 10^3^	3.92 × 10^3^	6.34 × 10^2^	1.38 × 10^4^
**Critical slowing down**	$\tau_{o}$ (s)	****	****	2.88 × 10^−9^	2.50 × 10^−12^
	$T_{g}$ (K)	****	****	58.39	205.55
	zυ	****	****	5.77	3.95
